# Unmet Home- and Community-Based Service Needs Among Informal Caregivers in Rural and Urban Areas

**DOI:** 10.1093/geroni/igaa057.1659

**Published:** 2020-12-16

**Authors:** Erin Bouldin, Jo-Ana Chase, Christina Miyawaki, David Russell, Nancy Gell, Christopher Taylor, Lisa McGuire

**Affiliations:** 1 Appalachian State University, Boone, North Carolina, United States; 2 University of Missouri, Columbia, Columbia, Missouri, United States; 3 University of Houston, Houston, Texas, United States; 4 The University of Vermont, Burlington, Vermont, United States; 5 Centers for Disease Control and Prevention, Atlanta, Georgia, United States

## Abstract

Home- and community-based services (HCBS) can reduce caregiver burden. We compared the prevalence of HCBS unmet needs among caregivers in rural and urban areas and identified factors associated with unmet HCBS needs. We used 2015-2018 Behavioral Risk Factor Surveillance System data, including the optional Caregiver Module, from 44 states, District of Columbia, and Puerto Rico. Caregivers were individuals providing care/assistance to a friend/family member with a long-term illness/disability during the past 30 days. Unmet needs were defined as needing, but not receiving, one or more of the following: caregiving classes, help accessing services, support groups, individual counseling, or respite care. “Rural” was defined as living outside Metropolitan Statistical Areas (available only for landline respondents). We calculated weighted estimates and used log-binomial regression to estimate adjusted prevalence ratios (PR). 19% of 25,180 caregivers lived in a rural area. Rural caregivers were less likely to report unmet HCBS needs (14.4% versus 20.6% urban, p<0.001), even after accounting for sociodemographic and caregiving characteristics (PR=0.81, 95% CI: 0.65-0.99, p=0.040). Unmet needs were more common among caregivers who provided more care, personal care, or care for someone with Alzheimer’s disease/dementia, regardless of rural residence. Although rural individuals can experience more barriers to accessing health services, rural caregivers in our study reported fewer unmet HCBS needs than urban caregivers. Additional research is needed to determine if stronger systems of informal support in rural areas may explain this difference. Further investigation of factors contributing to differences in unmet service needs among rural and urban caregivers is needed.

